# Erratum

**DOI:** 10.1111/jdi.12885

**Published:** 2018-09-04

The authors would like to draw the reader's attention to the error in the following article.

Yan WJ, Sun P, Wei DD, Wang SX, Yang JJ, Li YH, Zhang C. T cell immunoglobulin and mucin domain‐containing molecule 3 on CD14^+^ monocytes serves as a novel biological marker for diabetes duration in type 2 diabetes mellitus. *J Diabetes Investig* 2016; 7: 867–873.

On page 869, CD14^+^ T cells should have been replaced with CD14^+^ monocytes cells to read as follows in the legend of Figure 1.

**Figure 1** | T cell immunoglobulin and mucin domain‐containing molecule 3 (Tim‐3) expression on CD14^+^ monocytes cells in type 2 diabetes patients is significantly decreased. Peripheral blood mononuclear cells were isolated from healthy donors (*n* = 18) and patients with type 2 diabetes (*n* = 31). (a) Flow cytometry analysis of Tim‐3 expression on CD14^+^ monocytes cells (left) and the statistical graph is shown (right) for type 2 diabetes patients (*n* = 31, 30.43 ± 3.58%) and healthy donors (*n* = 18, 50.78 ± 2.36%). (b) Correlation analysis of Tim‐3 expression on CD14^+^ monocytes cells and type 2 diabetes patients’ fasting plasma glucose (FPG), glycated hemoglobin (HbA1c), insulin, body mass index (BMI), age and diabetes duration. ***P < 0.001.

The authors apologize for the error and any confusion it may have caused.

